# Testing extra-linearity across a psychosis continuum

**DOI:** 10.1186/s12888-021-03498-3

**Published:** 2021-11-16

**Authors:** Jeremy W. Coid, Yamin Zhang, Jinkun Zeng, Xiaojing Li, Qiuyue Lv, Wanjie Tang, Qiang Wang, Wei Deng, Wanjun Guo, Liansheng Zhao, Xiaohong Ma, Yajing Meng, Mingli Li, Huiyao Wang, Ting Chen, Min Yang, Tao Li

**Affiliations:** 1 Mental Health Center and Psychiatric Laboratory, the State Key Laboratory of Biotherapy, West China Hospital of Sichuan University, Chengdu, Sichuan China; 2 Institute of Emergency Management and Post-disaster Reconstruction, Sichuan University, Chengdu, Sichuan China; 3 Centre for Psychological Educational and Consultation, Sichuan University, Chengdu, Sichuan China; 4 Hangzhou Seventh People’s Hospital, Affiliated Mental Health Center, Zhejiang University School of Medicine, Hang zhou, Zhejiang China; 5 Department of Epidemiology and Health Statistics, West China School of Public Health, Sichuan University, Chengdu, Sichuan China; 6 West China Research Center for Rural Health Development, Sichuan University, Chengdu, Sichuan China

**Keywords:** Psychotic experiences, Psychosis continuum, Extra-linearity, Etiology, Psychosis subtypes

## Abstract

**Background:**

It is unclear whether psychotic experiences (PEs) gradually merge into states of clinical psychosis along a continuum which correspond to a dimensional classification or whether latent classes appear above a certain severity threshold which correspond better to diagnostic categories of psychosis.

**Methods:**

Annual cross-sectional surveys, 2014–19, among Chinese undergraduates (*N* = 47,004) measured PEs, depression and etiological risk factors using standardized self-report instruments. We created a psychosis continuum with five levels and tested linear and extra-linear contrasts in associated etiological risk factors, before and after adjustment for depression. We carried out latent class analysis.

**Results:**

Categorical expression of psychosis, including hallucinations and delusions, nuclear symptoms, and nuclear symptoms and depression were found at severe level 5. Etiological risk factors which impacted linearly across the continuum were more common for depression. Child maltreatment impacted extra-linearly on both psychosis and depression. Family history of psychosis impacted linearly on psychosis; male sex and urban birth impacted extra-linearly and were specific for psychosis. Four latent classes were found, but only at level 5. These corresponded to nuclear schizophrenia symptoms, nuclear schizophrenia and depressive symptoms, severe depression, and an unclassified category with moderate prevalence of PEs.

**Conclusion:**

Quantitative and qualitative changes in the underlying structure of psychosis were observed at the most severe level along a psychosis continuum, where four latent classes emerged. These corresponded to existing categorical classifications but require confirmation with clinical interview. PEs are non-specific and our findings suggest some are on a continuum with depression, whilst others are on a continuum with non-affective psychosis. Differing patterns of impact from etiological risk factors across the spectrum of psychopathology determine outcome at the most severe level of these continua.

**Supplementary Information:**

The online version contains supplementary material available at 10.1186/s12888-021-03498-3.

## Background

Psychotic experiences (PEs) are common in the general population and described as an extended phenotype of psychosis along a continuum, with clinical psychosis at the far end of severity [1]. If PEs gradually merge into states of clinical psychosis with no identifiable point of transition along the psychosis continuum, then dimensional diagnostic approaches are appropriate. However, if at a threshold of severity along the continuum there is both quantitative and qualitative underlying change in psychotic symptoms with appearance of latent classes [2, 3], this would support a categorical diagnostic approach. Binbay and colleagues [2] showed that increasing levels of severity along a “spectrum”, from PEs to psychotic symptoms to clinical psychosis, were influenced by a range of predictor variables. These included etiological factors and other non-psychotic psychopathological symptoms. Association between a particular predictor variable and position on the psychosis continuum could increase linearly, with increasing levels of impact of the predictor variable (continuity). Alternatively, there could be a disproportional shift up or down above the level of the predictor variable on the continuum (discontinuity). If there was a sudden shift upwards, this was positive extra-linear discontinuity, representing dramatic sudden increase in impact of the predictor variable, with underlying quantitative and additional qualitative changes in psychotic symptoms towards the most severe end of the continuum. If a sudden downwards trend, negative extra-linear discontinuity which indicates declining or absence of impact from the predictor variable towards the end of the continuum. If there were linear increase, this means graded increase in risk (continuity) and likely to be the outcome of additive effects of etiological risk factors resulting in increasingly severe, but correspondingly linear, presentation of psychosis. However, extra-linear increase means that threshold effects occur in the form of sudden increase in risk beyond a certain value discontinuity (see Figure S1 for diagrammatic representation of linearity and extralinearity). Extra-linearity, shown by dramatic increase in associations between psychotic symptoms and etiological factors at the severe end of a continuum has been described as a “quasi-continuous” relationship, explained by unmeasured moderators, with multifactorial etiology, and where multiple factors interact with each other [2, 4, 5]. Furthermore, there could be underlying changes in symptoms associated with extra-linearity which are not simply linear, with the hypothesized appearance of latent classes as distinct categorical entities. However, an association between both extra-linearity and appearance of latent classes along a psychosis continuum has not yet been confirmed.

### Are associations between predictors and PEs confounded by depression?

Although PEs are considered specific risk factors for transition to psychotic disorders such as schizophrenia, they are also non-specific, with risks for both psychotic and non-psychotic disorders [6, 7]. In the latter, PEs show no clear demarcation from symptoms of affective disorders [8–10] and can modify clinical and functional severity of depression (and other common mental disorders) resulting in poorer clinical course and functional outcome [11–13]. It has been argued that individuals with PEs are more likely to develop common mental disorders, including mood disorders, than psychotic disorder [7, 8]. PEs and affective symptoms could therefore show different quantitative and qualitative associations at different levels across a continuum. For example, a general population psychosis continuum showed dose-response linear relationships with both Manic and Depressive symptoms [2]. However, at the most severe level which included clinical cases of schizophrenia, linearity was no longer observed and associations with affective symptoms were considerably weaker [2]. This suggests there could be two different subtypes of psychosis: first, an extended psychosis phenotype associated with other non-psychotic conditions, primarily depression, where PEs and psychotic symptoms are closely associated with depressive symptoms across a continuum. Second, psychotic symptoms along a continuum with schizophrenia [14]. Associations between key risk factors and the latter subtype would only emerge following statistical adjustment for depressive symptoms because the associations would at first be confounded by the depressive symptoms.

Our aims were firstly, to describe the extended psychosis phenotype in our sample and model its relationship with depressive symptoms. Secondly, investigate associations between categorical constructs of psychosis, other non-psychotic psychopathology and 5 levels of severity of PEs across the continuum, before and after adjusting for depression. Thirdly, investigate linear and extra-linear associations between demography, categorical constructs of psychosis and depression, and putative etiological risk factors by identifying linearity and extra-linearity across the continuum, both before and after adjusting for depression. We finally aimed to identify whether latent classes of psychosis exist, independent from depression, at what level of psychosis symptom severity these emerge, and whether they corresponded to current classifications of psychosis to support existing categorical subtypes.

## Methods

We used a method previously pioneered by Binbay and colleagues [2] to investigate the full continuum of subclinical psychosis in a population of university students. We measured associations between continuous and categorical measures of PEs, other phenotypical expression of psychosis, and etiological risk factors. We further tested whether underlying latent categorical structures were identifiable across the continuum [2, 15]. Latent Class Analysis (LCA) classifies population heterogeneity into categorical groups of homogeneous individuals with implications for classification. In LCA, a group of people who are homogeneous in their symptom profile should present as a single latent class. More than one latent class would suggest multiple groups of individuals who are distinguishable based on their symptom profile and would be consistent with the need for different diagnostic categories to reflect these different symptom typologies [16]. We additionally tested whether our findings had been confounded by depressive symptoms associated with PEs.

### Participants

The Sichuan University Students Study is an ongoing investigation into mental health problems associated with student life, risk factors preceding university entry, and their impact on academic performance and mental health. All freshmen are invited annually to complete a questionnaire on-line, with a follow-up subsample at 1 year. The first year cross-sectional study sample was used for this investigation and included male and female respondents, 2014–2018. Students were asked to participate 1–3 months after university entry. After excluding those who gave incomplete information, 47,004 were included, an 83.9% response rate. More details can be found in our previous publications [17, 18].

### Measures

#### PEs

The Symptom Checklist-90-Revised (SCL-90-R) [19] assessed PEs in the past year. It has shown reliability among the Chinese general population [20] and university students [21]. The 16 variables were all moderately to strongly correlated with each other (Pearson’s correlation coefficient 0.24–0.57) and principal component analysis revealed a single component with eigenvalue greater than unity (6.47) explaining 40.4% of the variance. The variables were therefore combined into a single scale, 15 consisting of the sum score of the items, with good internal consistency (Crohnbachs alpha: .90). Two symptom dimensions relevant to psychosis include 10 items in the psychoticism and 6 items in the paranoia subscales. These were combined into a 16-item continuous measure of PEs as primary outcome. Scores ranged from 0 “not at all” to 4 “extremely” on a 5 point Likert scale. We created a psychosis continuum by dividing the population into 5 levels of PE severity. Since SCL-90-R scores followed half-normal distribution, a fold at the mean of an ordinary normal distribution with mean zero, we complemented the ordinary normal distribution by adding minus SCL-90-R score of all subjects. We created a continuum of PEs by setting scores of 0 (mean of the normal distribution dataset) as reference, then included one, two, three, and more than three standard deviations (SDs) which is 7.42 from 0 to create levels 2, 3, 4, and 5 respectively (see Supplementary materials).

We created two dummy variables, “Schizophrenia Nuclear Symptoms” and “Hallucinations and delusions” by including a selected number of items from the Psychoticism and Paranoid ideation sub-scales. To obtain these categorical psychosis measures of psychosis, we re-coded SCL-R-90 items as symptoms, present when scoring 2 (moderate) or above: (i) A categorical measure of Schizophrenia Nuclear Symptoms was created based on a previously developed SCL-R-90 sub-scale [8, 22]: ≥3 of 4 items rated ≥2 by Rossler and colleagues; (ii) Following the method of Binbay et al. [2], we created a dichotomous variable reflecting the combination of a hallucination with at least 1 delusion. “Hallucinations and Delusions” were present when Hearing Voices and two of four persecutory items in the Paranoid Ideation sub-scale were rated ≥2. More details of individual items used to create these dummy variables can be found in Supplementary materials.

#### Depression

The Patient Health Questionnaire-9 (PHQ-9) Depression module of the Prime-MD diagnostic instrument for common mental disorders measured Depressive symptoms over the past 2 weeks [23] with a categorical measure of probable Depressive disorder at 15 and above [24].

#### Psychiatric diagnosis

Participants were asked if they had ever consulted a medical practitioner and received a diagnosis of psychotic or non-psychotic mental disorder.

#### Etiological risk factors

Family income was rated at five levels ranging from < 5000 to > 100,000 RMB. Low family income was defined as below 10,000 Yuan annually. A total of 42,227 (89.8%) students were majority Han Chinese, with 840 (1.8%) Tujia, 440 (0.9%), Hui, 424 (0.9%) Miao, 370 (0.6%) Yi, 324 (0.7%) Man, 301 (0.6%) Tibetan, and 1812 (3.9%) from other ethnic minorities.

Participants were asked if first degree relatives had been diagnosed with severe (psychotic or non-psychotic) mental disorder. Birth place defined under China’s household registration was rated on six-levels from rural (countryside) to municipal city directly under the Central Government [25]. Urban birth was rated at provincial capital city level 5 and municipal city 6.

Participants self-reported childhood adversities using Childhood Section of the Chinese World Mental Health Initiative Composite International Diagnostic Interview [26, 27], including loss of parent through divorce or death, experience of physical, sexual abuse, or neglect before 16.

### Statistical analysis

The relationship between PEs and depressive symptoms was investigated using Spearman correlation across the entire continuum and five levels.

Associations between 5 levels of continuum and other variables were expressed as β derived from linear regression for continuous variables or OR derived from logistic regression for dichotomous variables. Our reference level 1 was no PE scores on SCL-90-R. Continuum was modelled as independent variable. It should not make any difference whether continuum is the dependent or independent variables because this is a cross-sectional study.

**Fig. 1:**
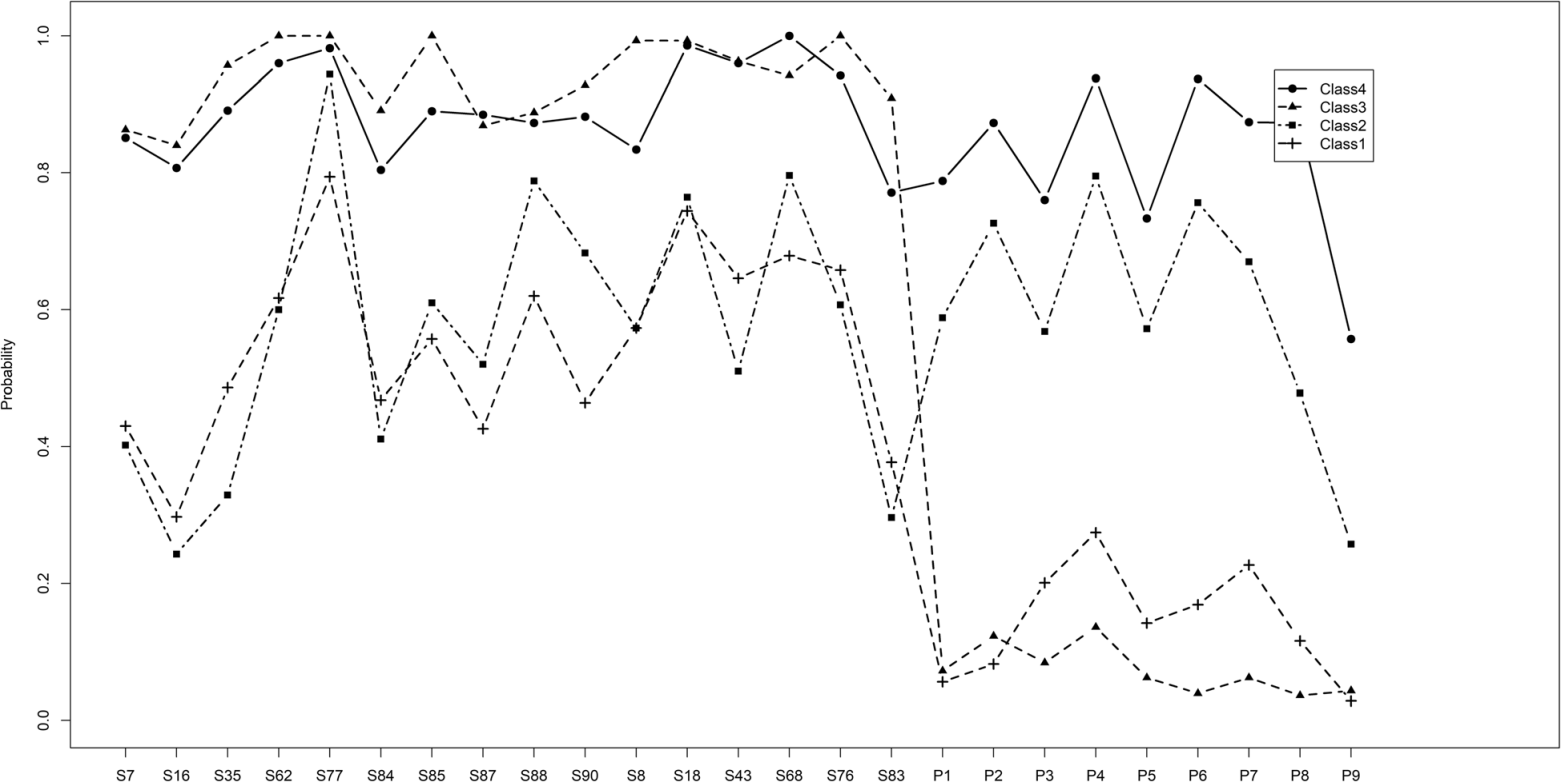
.ᅟ

The layout of the results in the tables is as follows. For each psychopathological (Table 2), or putative etiological (Table 3) variable, results were depicted in the corresponding table row, showing (1) the OR for association with the 5 groups of the spectrum variable (with absence or lowest level of PEs as the reference group, i.e. OR = 1), (2) whether or not the association deviated from linearity and, if so, deviation was positive or negative (Fig. 1), and (3) the test for significance for deviation from linearity.

To identify homogeneous subgroups, LCA were carried out using items in SCL-R-90 and PHQ-9 in the total sample and at different levels for subsamples. LCA is often used to identify subgroups that have a given probability of occurrence and are characterized by a specific and predictable combination of the analysed features. It derives classes using a formal probabilistic approach. The optimal number of classes is one that minimizes the degree of relationship among cases belonging to different classes [28].

Models with 2 to 6 classes were compared for each dataset first then a model with more classes was fitted if necessary. We used maximum likelihood as the estimator and all LCA models were estimated with 50 random starts at the initial stage and 5 optimisations at the final stage; if the log-likelihood could not be replicated (an indication of local maxima), we increased the parameters to 2000 and 200 respectively. The best solution is the solution with the largest log-likelihood.

Selection of optimal number of latent classes was based on information criteria, Lo-Mendell-Rubin (LMR) test, and entropy. More details can be found in the supplementary materials. We finally drew a plot of model probabilities of response for each item, where y axis is the probability that people within LC endorse a specific item (score ≥ 2). To characterize the relationship between each class and psychopathological, etiological variables, logistic regression was used with classes as independent variable.

LCA analyses were carried out in Mplus 7.4. All other analyses were carried out using R3.3.2. Bonferroni’s correction was applied to correct multiple comparisons.

## Results

### Modelling PEs and depressive symptoms across the continuum

Mean age of the student population was 18.12 years (SD 0.91), 49.9% were male, most Han Chinese (89.8%), with family backgrounds having high or medium level earnings (85.1%). Table 1 shows distribution of PEs and Depressive symptoms across the continuum. Depressive symptoms showed a dose-response linear relationship with increasing severity of PEs across each level. Fig. S2 shows this linear relationship but also that the mean score of PEs was not entirely linear following standardization, with increase in levels 4 and 5. Although PEs and Depressive symptoms were highly correlated across the entire continuum, ρ = 0.64 (0.63–0.65) using Spearman rho, Table 1 and Fig. S2 show non-linear, U-shaped, underlying patterns of correlation across levels of severity. Correlations declined linearly from level 1 to 4, followed by a rise in correlation at level 5.

**Table 1:** Correlation of Psychotic Experiences and Depression for level of outcome across five levels (*n* = 47,004)

	PEs level1 *N* = 13,978 (29·7%)		PEs level2 *N* = 23,567 (50·1%)		PEs level3 *N* = 6402 (13·6%)		PEs level4 *N* = 2118 (4·5%)		PEs level5 *N* = 939 (2·0%)	
	Mean (SD)	*β*	Mean (SD)	*β (*95%CI)	Mean (SD)	*β* (95%CI)	Mean (SD)	*β* (95%CI)	Mean (SD)	*β* (95%CI)
Psychotic Experiences (SCL90-R) 4·44 (SD 5·95)	0 (0)	0·00 (Ref)	3·29 (1·92)	3·29***^$^ (3·25–3·33)	10·31 (1·94)	10·31***^$^ (10·25–10·37)	17·55 (2·15)	17·55***^$^ (17·46–17·64)	29·75 (7·26)	29·75***^$^ (29·62–29·87)
Depressive Symptoms (PHQ 9) 3·00 (SD 3·42)	0·92 (1·83)	0·00 (Ref)	2·97 (2·52)	1·88***^$^ (1·82–1·93)	5·52 (3·42)	4·61***^$^ (4·53–4·69)	7·77 (4·23)	6·86***^$^ (6·73–6·98)	11·11 (5·66)	10·19***^$^ (10·01–10·37)
Rho (95%CI)^a^		–		0·35**^@^ (0·34, 0·36)		0·20*^@^ (0·18, 0·23)		0·10* (0·06, 0·15)		0·25* (0·19, 0·31)

^a^Spearman correlation was performed, where rho is the correlation coefficient and CI is the confidence interval

*p < 0·05, **p < 0·01, ***p < 0·001

^$^: significance after correction for regressions of 21 variables using Bonferroni’s correction and the new threshold is 0.05/21 = 0.0024

^@^: significance after correction for 4 correlations using Bonferroni’s correction and the new threshold is 0.05/21 = 0.0125

### Categorical measures of psychosis, depression, and non-psychotic psychopathology

Table 2 shows distribution of categorical measures of psychosis phenotypes, depression, and clinical diagnoses across the continuum. Hallucinations and Delusions, Nuclear symptoms, and Nuclear symptoms and depression could not be tested for extra-linearity but showed substantial increase in odds of association at level 5. There was no attenuation following adjustment for depression which resulted in increased odds of association between level and each of these three phenotypes. Clinical diagnoses of Schizophrenia and non-psychotic disorder showed a dose-response linear relationship, together with positive extra-linear associations across the continuum. Adjustment for depression measured using the PHQ-9 resulted in attenuation. Positive extra-linearity was also found for depressive disorder across the continuum.

**Table 2:** Distribution of Categorical measures of Psychosis Phenotypes, Depression, and Clinical diagnosis for level of outcome (*n* = *n* = 47,004)

	PEs level1 *N* = 13,978 (29·7%)		PEs level2 *N* = 23,567 (50·1%)		PEs level3 *N* = 6402 (13·6%)		PEs level4 *N* = 2118 (4·5%)		PEs level5 *N* = 939 (2·0%)		Deviation From Linearity		
	N (%)	OR	N (%)	OR (95%CI)	N (%)	OR (95%CI)	N (%)	OR (95%CI)	N (%)	OR (95%CI)	*β*^a^	χ^**2**^	*P*-value
Hallucinations and Delusions *n* = 324 (0·7%)	0 (0·0)	–	0 (0·0)	–	23 (0·4)	1·00 (Ref)	87 (4·1)	11·94***^$^ (7·52–18·96)	214 (22·8)	82·52***^$^ (53·29–127·77)	–	–	–
		–		–		1·00 (Ref)		12·98***^$^ (8·15–20·68)		101·83***^$^ (64·49–160·77)	–	–	–
Schizophrenia Nuclear Symptoms *n* = 360 (0·8%)	0 (0·0)	–	0 (0·0)	–	10 (0·2)	1·00 (Ref)	48 (2·3)	14·87***^$^ (7·51–29·44)	302 (32·2)	303·68***^$^ (160·88–573·21)	–	–	–
		–		–		1·00 (Ref)		7·10***^$^ (3·74–13·48)		127·81***^$^ (72·28–225·98)	–	–	–
Nuclear Symptoms and Depression *n* = 85 (0·2%)	0 (0·0)	–	0 (0·0)	–	0 (0·0)	–	3 (0·1)	1·00 (Ref)	82 (8·7)	67·55***^$^ (21·28–214·37)	–	–	–
		–		–		–		1·00 (Ref)		26·56***^$^ (8·18–86·21)	–	–	–
Depressive Disorder *n* = 567 (1·2%)	24 (0·2)	1·00 (Ref)	50 (0·2)	1·24 (0·76–2·01)	103 (1·6)	9·57***^$^ (6·13–14·94)	144 (6·8)	42·83***^$^ (27·73–66·16)	246 (26·2)	208·41***^$^ (136·07–319·20)	0·58	166·12	< 0·001^$^
Diagnosed Schizophrenia *n* = 63 (0·1%)	10 (0·07)	1·00 (Ref)	19 (0·08)	1·17 (0·54–2·52)	12 (0·2)	2·73* (1·18–6·32)	8 (0·4)	5·48***^$^ (2·16–13·90)	14 (1·5)	22·16***^$^ (9·80–50·10)	0·43	22·06	< 0·001^$^
		1·00 (Ref)		0·95 (0·44–2·06)		1·59 (0·66–3·86)		2·41 (0·86–6·74)		6·19***^$^ (2·11–18·17)	0·32	9·98	0·002^$^
Diagnosed Non-Psychotic Disorder *n* = 2321 (4·9%)	258 (1·8)	1·00 (Ref)	907 (3·8)	2·16***^$^ (1·88–2·48)	604 (9·4)	5·61***^$^ (4·83–6·51)	332 (15·7)	9·99***^$^ (8·43–11·85)	220 (23·4)	16·52***^$^ (13·59–20·08)	0·14	51·13	< 0·001^$^
		1·00 (Ref)		1·76***^$^ (1·52–2·03)		3·37***^$^ (2·87–3·96)		4·69***^$^ (3·87–5·68)		5·36***^$^ (4·23–6·80)	0·05	6·80	0·009

Adjusted for age and sex· Second row for each variable additionally adjusted for PHQ-9 score

*p < 0·05, **p < 0·01,***p < 0·001

^a^regression coefficient for the squared term of PEs scores· A negative squared term suggests a qualitatively stronger association at the lower end of the PEs spectrum, and a positive squared term suggests a qualitatively stronger association at the higher end of the PEs spectrum. Significant deviation from linearity indicates a positive linear effect

^$^: significance after correction for regressions of 21 variables using Bonferroni’s correction and the new threshold is 0.05/21 = 0.0024

### Etiology and psychosis continuity

Table 3 shows distribution of etiological and demographic variables. Younger age showed dose-response associations across the continuum which increased after adjustment. However, adjustment resulted in extra-linearity in association with age being no longer being significant. A dose-response association between psychosis and male sex emerged after adjustment, with positive extra-linear association. A dose-response relationship between ethnic minority status was observed until level 4. Linearity was no longer observed following adjustment. Similarly, low family income initially showed a dose-response relationship and positive extra-linearity, but neither relationship was observed following adjustment.

**Table 3:** Distribution of putative Etiological Variables for level of outcome (*n* = 47,004)

	PEs level1 *N* = 13,978 (29·7%)		PEs level2 *N* = 23,567 (50·1%)		PEs level3 *N* = 6402 (13·6%)		PEs level4 *N* = 2118 (4·5%)		PEs level5 *N* = 939 (2·0%)		Deviation From Linearity		
	Mean (SD)	*β*	Mean (SD)	*β* *(*95%CI)	Mean (SD)	*β* *(*95%CI)	Mean (SD)	*β* *(*95%CI)	Mean (SD)	*β* *(*95%CI)	*β*^a^	**χ**^**2**^	*P*-value
Demography													
Age 18·19 (SD 0·91)	18·23 (0·95)	0·00 (Ref)	18·18 (0·90)	−0·05***^$^ (− 0·06−− 0·03)	18·16 (0·88)	− 0·06***^$^ (− 0·09−− 0·04)	18·16 (0·87)	−0·07**^$^ (− 0·11–0·02)	18·17 (0·83)	−0·06 (− 0·12–0·00)	0·01	4·30	0·03
		0·00 (Ref)		−0·07 ***^$^ (− 0·09−− 0·05)		−0·12***^$^ (− 0·15−− 0·09)		-0·15***^$^ (− 0·19--0·10)		-0·18***^$^ (− 0·24--0·11)	0·00	0·01	0·93
	N (%)	OR	N (%)	OR (95%CI)	N (%)	OR (95%CI)	N (%)	OR (95%CI)	N (%)	OR (95%CI)			
Male *n* = 23,444 (49·9%)	7353 (52·6)	1·00 (Ref)	11,313 (48·0)	0·83***^$^ (0·80–0·87)	3213 (50·2)	0·91**^$^ (0·86–0·96)	1096 (51·7)	0·97 (0·88–1·06)	469 (49·9)	0·90 (0·79–1·03)	0·06	42·18	< 0·001^$^
		1·00 (Ref)		0·91***^$^ (0·91–0·95)		1·12***^$^ (1·05–1·20)		1·32***^$^ (1·19–1·47)		1·43***^$^ (1·23–1·66)	0·10	98·30	< 0·001^$^
Ethnic Minority *n* = 4777 (10·1%)	1358 (9·7)	1·00 (Ref)	2375 (10·1)	1·05 (0·98–1·13)	693 (10·8)	1·15** (1·04–1·26)	248 (11·7)	1·26**^$^ (1·09–1·45)	103 (11·0)	1·16 (0·94–1·44)	0·02	1·69	0·19
		1·00 (Ref)		0·99 (0·92–1·07)		0·99 (0·89–1·11)		1·01 (0·86–1·19)		0·84 (0·66–1·07)	0·00	0·01	0·91
Low Family Income *n* = 6986 (14·9%)	2047 (14·6)	1·00 (Ref)	3446 (14·6)	1·02 (0·96–1·08)	970 (15·1)	1·06 (0·98–1·16)	339 (16·0)	1·14* (1·00–1·29)	184 (19·6)	1·46***^$^ (1·23–1·73)	0·04	8·72	0·003
		1·00 (Ref)		0·95 (0·89–1·01)		0·89* (0·81–0·98)		0·87 (0·75–1·00)		0·98 (0·80–1·19)	0·01	0·36	0·55
Urban Birth *n* = 6791 (14·4%)	2292 (16·4)	1·00 (Ref)	3221 (13·7)	0·80***^$^ (0·75–0·85)	835 (13·0)	0·76***^$^ (0·69–0·82)	318 (15·0)	0·89 (0·79–1·01)	125 (13·3)	0·78* (0·64–0·94)	0·06	16·68	< 0·001^$^
		1·00 (Ref)		0·87***^$^ (0·81–0·92)		0·92 (0·84–1·01)		1·19* (1·03–1·38)		1·19 (0·96–1·48)	0·09	37·27	< 0·001^$^
	PEs level1 *N* = 13,978 (29·7%)		PEs level2 *N* = 23,567 (50·1%)		PEs level3 *N* = 6402 (13·6%)		PEs level4 *N* = 2118 (4·5%)		PEs level5 *N* = 939 (2·0%)		Deviation From Linearity		
	N (%)	OR	N (%)	OR (95%CI)	N (%)	OR (95%CI)	N (%)	OR (95%CI)	N (%)	OR (95%CI)	*β*^a^	**χ**^**2**^	*P*-value
Family History													
Family History (psychosis) *n* = 338 (0·7%)	67 (0·5)	1·00 (Ref)	159 (0·7)	1·40* (1·05–1·86)	69 (1·1)	2·25***^$^ (1·60–3·15)	27 (1·3)	2·67***^$^ (1·70–4·18)	16 (1·7)	3·58***^$^ (2·06–6·19)	0·05	1·10	0·29
		1·00 (Ref)		1·26 (0·94–1·69)		1·74** (1·20–2·54)		1·82* (1·09–3·05)		2·02* (1·03–3·94)	0·10	0·03	0·86
Family History (non-psychosis) *n* = 440 (0·9%)	79 (0·6)	1·00 (Ref)	214 (0·9)	1·58***^$^ (1·22–2·05)	87 (1·4)	2·39***^$^ (1·76–3·25)	50 (2·3)	4·22***^$^ (2·95–6·03)	10 (1·1)	1·87 (0·97–3·62)	-0·01	0·01	0·91
		1·00 (Ref)		1·42** (1·09–1·85)		1·82***^$^ (1·29–2·55)		2·79***^$^ (1·83–4·25)		1·00 (0·48–2·11)	-0·05	1·06	0·30
Adverse Childhood Experiences													
Loss of Parent (Divorce) *n* = 1102 (2·3%)	191 (1·3)	1·00 (Ref)	546 (2·3)	1·71***^$^ (1·45–2·02)	219 (3·4)	2·57***^$^ (2·11–3·12)	85 (4·0)	3·03***^$^ (2·34–3·94)	61 (6·0)	5·03***^$^ (3·74–6·77)	0·03	0·93	0·34
		1·00 (Ref)		1·61***^$^ (1·36–1·91)		2·21***^$^ (1·78–2·74)		2·42***^$^ (1·80–3·25)		3·59***^$^ (2·50–5·16)	0·00	0·01	0·92
Loss of Parent (Death) *n* = 632 (1·3%)	145 (1·0)	1·00 (Ref)	304 (1·3)	1·25* (1·02–1·53)	114 (1·8)	1·74***^$^ (1·36–2·23)	48 (2·2)	2·23***^$^ (1·60–3·10)	21 (2·2)	2·19***^$^ (1·38–3·49)	0·06	2·41	0·12
		1·00 (Ref)		1·16 (0·94–1·42)		1·44** (1·09–1·90)		1·68** (1·15–2·45)		1·43 (0·83–2·46)	0·03	0·55	0·46
Physical Abuse *n* = 14,785 (31·5%)	3829 (27·4)	1·00 (Ref)	6773 (28·7)	1·09***^$^ (1·04–1·14)	2625 (41·0)	1·88***^$^ (1·76–2·00)	1019 (48·1)	2·49***^$^ (2·27–2·74)	539 (57·4)	3·66***^$^ (3·20–4·19)	0·17	253·88	< 0·001^$^
		1·00 (Ref)		1·01 (0·96–1·06)		1·55***^$^ (1·44–1·66)		1·87***^$^ (1·68–2·08)		2·40***^$^ (2·06–2·80)	0·14	171·46	< 0·001^$^
Sexual Abuse *n* = 1360 (2·9%)	311 (2·2)	1·00 (Ref)	666 (2·8)	1·25**^$^ (1·09–1·43)	208 (3·2)	1·46***^$^ (1·22–1·75)	102 (4·8)	2·22***^$^ (1·77–2·79)	73 (7·8)	3·67***^$^ (2·82–4·78)	0·13	25·67	< 0·001^$^
		1·00 (Ref)		1·12 (0·97–1·29)		1·11 (0·91–1·35)		1·46** (1·13–1·90)		1·97***^$^ (1·42–2·74)	0·08	9·50	0·002^$^
Neglect *n* = 16,056 (34·2%)	3511 (25·1)	1·00 (Ref)	7707 (32·7)	1·46***^$^ (1·39–1·53)	3042 (45·7)	2·72***^$^ (2·55–2·89)	1198 (56·6)	3·90***^$^ (3·55–4·29)	598 (63·9)	5·27***^$^ (4·59–6·05)	0·12	128·09	< 0·001^$^
		1·00 (Ref)		1·32***^$^ (1·25–1·38)		2·11***^$^ (1·97–2·27)		2·69***^$^ (2·42–2·99)		3·06***^$^ (2·62–3·57)	0·09	64·77	< 0·001^$^
> =2 maltreatment *n* = 10,567 (22.5%)	2991 (21.4)	1.00 (Ref)	4531 (19.2)	0.88***^$^ (0.84–0.93)	1811 (28.3)	1.46***^$^ (1.36–1.56)	790 (37.3)	2.20***^$^ (1.99–2.42)	444 (47.3)	3.33***^$^ (2.91–3.81)	0.24	434	< 0·001^$^
		1.00 (Ref)		0.82***^$^ (0.77–0.86)		1.20***^$^ (1.11–1.29)		1.64***^$^ (1.47–1.83)		2.17***^$^ (1.85–2.53)	0.21	315.23	< 0·001^$^

No adjustment for demographic variables· Adjusted for age and sex in the first row for the rest variables· Second row for each variable additionally adjusted for PHQ-9 score

*P < 0·05, **p < 0·01, ***p < 0·001

^a^regression coefficient for the squared term of PEs scores· A negative squared term suggests a qualitatively stronger association at the lower end of the PEs spectrum, and a positive squared term suggests a qualitatively stronger association at the higher end of the PEs spectrum. Significant deviation from linearity indicates a positive linear effect

^$^: significance after correction for regressions of 21 variables using Bonferroni’s correction and the new threshold is 0.05/21 = 0.0024

There was a dose-response association with Family History of psychosis across the continuum. This remained following adjustment, but with attenuation. There was a dose-response relationship between family history of non-psychotic disorder until level 4, but this association was not significant at level 5. This pattern remained, with attenuation, after adjustment.

There was a dose-response relationship with urban birth until level 4 but with negative odds of association across the continuum. Following adjustment, these odds of association were increased positively rather than attenuated. This association across the continuum showed positive extra-linearity which showed increase after adjustment for depression.

Loss of parent through divorce showed a dose-response association with attenuation after adjustment. Loss of parent through death showed a linear relationship until level 4 which was attenuated following adjustment. For adverse childhood experiences of physical and sexual abuse and neglect, dose-response linear relationships remained, together with positive extra-linear associations following adjustment, although each showed some reduction in extra-linearity following adjustment.

### Latent classes

Table 4 shows results of fitting LC models with different numbers of classes to binary SCL90-R symptoms. No LC solution could be found for either the entire sample or between levels 1–4. However, in level 5 a 4-class solution can be selected (Fig. 1). LMRT and entropy suggested a 4-class model. Although BIC suggested a 5-class model, adjusted BIC kept decreasing as the number of classes increasing. Therefore, a 4-class solution was selected.

**Table 4:** Goodness of fit for latent class models (*n* = 47,004)

Number of classes	Log Likelihood	Number of parameters	AIC	BIC	AdjBIC	LMRT P-value	Entropy	BLRT *P*-value	No. classes with *n* < 5% study sample
Level 1–5									
2	− 156,499	51	313,100	313,547	313,385	0.33	0.94	0.00	0
3	−151,298	77	302,751	303,425	303,180	0.00	0.90	0.00	1
4	−148,819	103	297,844	298,746	298,419	0.00	0.91	0.00	1
5	− 147,881	129	296,016	297,146	296,736	0.68	0.90	0.00	2
6	−147,078	155	294,467	295,824	295,332	0.00	0.89	0.00	4
Level 1–4									
2	−133,617	51	267,337	267,783	267,620	0.00	0.90	0.00	0
3	−130,798	77	261,750	262,423	262,178	0.00	0.89	0.00	1
4	− 129,473	103	259,153	260,053	259,726	0.00	0.88	0.00	2
5	−129,004	129	258,267	259,394	258,984	0.68	0.85	0.00	3
6	− 128,604	155	257,519	258,874	258,381	0.10	0.86	0.00	5
Level 5									
2	−13,929	51	27,960	28,208	28,046	0.00	0.83	0.000	0
3	−13,748	77	27,651	28,024	27,780	0.0004	0.85	0.000	0
4	−13,579	103	27,365	27,864	27,537	**0.001**	**0.87**	0.000	0
5	−13,456	129	27,170	**27,795**	27,386	0.11	0.83	0.000	0
6	−13,389	155	27,088	27,839	27,347	0.72	0.78	0.000	1

For binary responses, SCL-90-R (cutoff = 2); PHQ9 (cutoff = 2)

*LMRT* Lo-Mendell-Rubin test

*BLRT* Bootstrap Likelihood Ratio Test

Numbers in bold are indicative when selecting the best model

Figure 1 shows the four latent classes found at level 5. Class 1 was the largest (*N* = 490, 52.2%) and characterized by moderately high prevalence of SCL-R-90 items but low prevalence of PHQ-9 depressive symptoms and was classified as a High Risk state for psychosis along a spectrum with schizophrenia but not meeting criteria for the latter in terms of level of severity of psychosis. Class 2 (*N* = 337, 35.9%) was characterized by both moderately high SCL-R-90 items and depressive symptoms and was classified as moderate-severe Clinical depression. Class 3 (*N* = 51, 5.4%) by high SCL-90-R items and lowest prevalence of depressive symptoms and was classified as showing prototypical symptoms and clusters of symptoms of Schizophrenia. Class 4 (*N* = 61 6.5%) by both high prevalence of SCL-R-90 items and depressive symptoms and was classified as Schizophrenia/Depression showing features that could be considered indicative of schizoaffective disorder or alternatively schizophrenia with co-morbid depressive episode. Table S1 further discriminates between the four classes by comparing prevalence of SCL-R-90 items and depressive symptoms with Class 1 as reference. Higher prevalence of most depressive symptoms were found for classes 2 and 4, and SCL-90-R items for classes 3 and 4, also shown in Fig. 1. However, when comparing classes 1 and 2, Class 1 showed higher prevalence for hearing voices, believing others aware of thoughts, being watched or talked about, and people taking advantage, corresponding to a high risk state on a spectrum with schizophrenia. Class 2 showed lower prevalence for lonely even with people, something wrong with body, never feeling close to another person, and something wrong with their mind, corresponding to Depressive disorder.

Table 5 shows higher mean scores for SCL-90-R items in Classes 2, 3 and 4 than Class1, with highest scores in Class 4. Hallucinations and delusions and nuclear symptoms were less prevalent in Class 2 than 1 and more prevalent in Classes 3 and 4. Nuclear symptoms and depression was mainly found in Class 4. There were no cases in either Class 1 or 3 that met the threshold for a diagnosis of depression using PHQ-9. There were no differences between classes in prevalence of a clinical diagnosis of schizophrenia, although classes 2 and 4 showed higher prevalence of clinical diagnosis of non-psychotic disorder.

**Table 5:** Comparison of SCL-90-R score, psychosis phenotype, and clinical diagnosis between Classes (*n* = 939)

	Class 1 (High Risk) *N* = 490 (52.2%)	Class 2 (Depression) *N* = 337 (35.9%)	Class 3 (Prototypical Scizophrenia) *N* = 51 (5.4%)	Class 4 (Schizophrenia/Depression) *N* = 61 (6.5%)	Class 1 β(95%CI)	Class 2 β(95%CI)	Class 3 β(95%CI)	Class 4 β(95%CI)	Class 4 vs.3 β(95%CI)
Psychotic Experiences (SCL-90-R)	27.40 ± 4.45	29.30 ± 5.45	39.67 ± 10.35	42.80 ± 10.17	0 (ref)	1.91***^$^ (1.11–2.72)	12.26***^$^ (10.6–13.93)	15.42***^$^ (13.88–16.97)	2.93 (−0.9–6.76)
Paranoid ideation subscale	11.00 ± 2.96	11.12 ± 3.39	15.18 ± 4.15	16.15 ± 4.18	0 (ref)	0.10 (−0.35–0.56)	4.18***^$^ (3.23–5.12)	5.13***^$^ (4.25–6.00)	0.85 (− 0.70–2.39)
Psychoticism subscale	16.40 ± 3.56	18.18 ± 4.23	24.49 ± 6.48	26.66 ± 6.70	0 (ref)	1.81***^$^ (1.22–2.40)	8.09***^$^ (6.86–9.32)	10.29***^$^ (9.16–11.43)	2.08 (− 0.39–4.56)
					Class 1 **OR(95%CI)**	Class 2 **OR (95%CI)**	Class 3 **OR (95%CI)**	Class 4 **OR (95%CI)**	Class 4 vs.3 **OR (95%CI)**
Hallucinations and Delusions	115 (23.5%)	38 (11.3%)	27 (52.9%)	34 (55.7%)	1 (ref)	0.40***^$^ (0.27–0.60)	3.71***^$^ (2.05–6.72)	3.93***^$^ (2.26–6.85)	1.07 (0.50–2.27)
Schizophrenia nuclear symptoms	131 (26.7%)	66 (19.6%)	50 (98.0%)	55 (90.2%)	1 (ref)	0.65* (0.47–0.92)	144.17***^$^ (19.68–1056.25)	26.35***^$^ (11.03–62.96)	0.21 (0.02–1.84)
Nuclear symptoms and Depression	0 (0%)	37 (11%)	0 (0%)	45 (73.8%)	1 (ref)	n.a.	n.a.	n.a.	n.a.
Depression (cutoff)	0 (0%)	195 (57.9%)	0 (0%)	51 (83.6%)	1 (ref)	n.a.	n.a.	n.a.	n.a.
Diagnosed Schizophrenia	4 (0.8)	7 (2.1)	1 (2.0)	2 (3.3)	1 (ref)	2.57 (0.74–8.92)	2.14 (0.23–19.97)	3.13 (0.53–18.53	1.38 (0.11–17.44)
Diagnosed Non-Psychotic Disorder	90 (18.4)	99 (29.4)	11 (21.6)	20 (32.8)	1 (ref)	1.84***^$^ (1.33–2.56)	1.21 (0.60–2.45)	2.09* (1.17–3.75)	1.72 (0.71–4.16)

Adjusted for age and sex

^$^: significance after correction for regressions of 21 variables using Bonferroni’s correction and the new threshold is 0.05/21 = 0.0024

Table 6 shows discriminating demographic and etiological risk factors. Classes 2 and 4 were more likely from an ethnic minority. Class 4 was more likely to have low family income, to be older, and report sexual abuse in childhood. Classes 2 and 4 were more likely to have experienced physical abuse and neglect.

**Table 6:** Comparison of Demography and putative Etiological Risk Factor between Classes (*n* = 939)

	Class 1 (High Risk) *N* = 490 (52.2%)	Class 2 (Depression) *N* = 337 (35.9%)	Class 3 (Prototypical Schizophrenia) *N* = 51 (5.4%)	Class 4 (Schizophrenia/Depression) *N* = 61 (6.5%)	Class 1 OR (95%CI)	Class 2 OR (95%CI)	Class 3 OR (95%CI)	Class 4 OR (95%CI)	Class 4 vs.3 OR (95%CI)
Male	252 (51.4%)	157 (46.6%)	28 (54.9%)	32 (52.5%)	1 (ref)	0.82 (0.62–1.09)	1.15 (0.64–2.05)	1.04 (0.61–1.78)	0.91 (0.43–1.91)
Ethnic minority	56 (11.4%)	38 (11.3%)	4 (7.8%)	5 (8.2%)	1 (ref)	35.59***^$^ (23.51–53.87)	1.56 (0.63–3.90)	83.76***^$^ (36.7–191.16)	0.84 (0.31–2.31)
Low family income	83 (16.9%)	70 (20.8%)	13 (25.5%)	18 (29.5%)	1 (ref)	1.29 (0.90–1.83)	1.68 (0.86–3.29)	2.05* (1.13–3.74)	1.22 (0.53–2.82)
Family History (psychosis)	6 (1.2%)	7 (2.1%)	1 (2.0%)	2 (3.3%)	1 (ref)	1.65 (0.55–4.99)	1.66 (0.19–14.12)	3.06 (0.60–15.68)	1.74 (0.15–20.52)
Family History (non-psychosis)	3 (0.6%)	7 (2.1%)	0 (0%)	0 (0%)	1 (ref)	3.37 (0.86–13.17)	0 (0-Inf)	0 (0-Inf)	1 (0-Inf)
Urban birth	72 (14.7%)	39 (11.6%)	10 (19.6%)	4 (6.6%)	1 (ref)	0.76 (0.50–1.15)	1.43 (0.69–2.99)	0.42 (0.15–1.19)	0.28* (0.08–0.97)
Loss of parent (divorce)	31 (6.3%)	24 (7.1%)	2 (3.9%)	4 (6.6%)	1 (ref)	1.13 (0.65–1.97)	0.59 (0.14–2.54)	0.97 (0.33–2.87)	1.75 (0.3–10.01)
Loss of parent (death)	12 (2.4%)	7 (2.1%)	1 (2.0%)	1 (1.6%)	1 (ref)	0.83 (0.32–2.14)	0.79 (0.10–6.22)	0.60 (0.08–4.79)	0.89 (0.05–15.71)
Physical abuse	262 (53.5%)	204 (60.5%)	30 (58.8%)	43 (70.5%)	1 (ref)	1.36* (1.03–1.81)	1.22 (0.68–2.20)	2.04* (1.14–3.66)	1.75 (0.79–3.90)
Sexual abuse	26 (5.3%)	27 (8.0%)	6 (11.8%)	14 (23.0%)	1 (ref)	1.53 (0.87–2.67)	2.41 (0.94–6.18)	5.09***^$^ (2.47–10.49)	2.17 (0.76–6.21)
Neglect	279 (56.9%)	244 (72.4%)	31 (60.8%)	44 (72.1%)	1 (ref)	1.99***^$^ (1.47–2.68)	1.16 (0.64–2.10)	1.91* (1.06–3.44)	1.72 (0.77–3.84)
					Class 1 **β (95%CI)**	Class 2 **β (95%CI)**	Class 3 **β (95%CI)**	Class 4 **β (95%CI)**	
Age	18.14 ± 0.81)	18.18 ± 0.81	18.20 ± 0.80	18.38 ± 1.05	0 (ref)	0.04 (−0.07–0.16)	0.06 (−0.18–0.30)	0.24* (0.02–0.46)	0.18 (−0.17–0.53)

Adjusted for age and sex

^$^: significance after correction for regressions of 21 variables using Bonferroni’s correction and the new threshold is 0.05/21 = 0.0024

When comparing Classes 3 and 4, these were differentiated by associations with depressive symptoms (Table S1) and by urban birth (Table 6).

## Discussion

The underlying structure of psychosis demonstrated marked quantitative and qualitative change at level 5 along a continuum of psychosis. Psychotic symptoms initially showed a pattern of increase resembling a simple dimension (continuity). However, with increasing severity towards a threshold at which hallucinations and delusions, nuclear symptoms, and clinical schizophrenia were observed, there was both extra-linearity in associated symptoms of depression and the impact from certain etiological risk factors (discontinuity). Once this level was reached, natural boundaries began to emerge between symptom clusters, and finally four LCs were observed. These underlying changes are further demonstrated in correlations observed between SCL-90-R and PHQ-9 items at each level, with progressive decline followed by dramatic reversal at level 5. Depressive symptoms showed dose-response increases with each level of increasing PE severity, initially suggesting no clear demarcation from PEs, and corresponding to previous studies [8–13, 29–31]. However, these findings concealed more complex underlying patterns and inter-relationships between PEs and Depression across the continuum. These demonstrated that whilst both showed a linear increase, their inter-relationship across the continuum was in fact non-linear.

At the most severe level 5, heterogeneity was observed. Some participants at level 5 showed high levels of PEs but low levels of Depression (Classes 1 and 3). Declining correlations between PEs and Depressive items until level 4 suggested they were becoming increasingly independent of each other until level 5 where this process showed some reversal. The most likely explanation was that two latent classes had emerged at level 5 in which psychotic symptoms and depressive symptoms were still closely associated (Classes 2 and 4), and corresponding to the extended psychosis phenotype associated with depression. The other classes (1 and 3) included psychotic symptoms and few depressive symptoms, possibly along a continuum with schizophrenia, but not depression.

A further key finding was that LCs could not be found when using either the entire sample or if LCA was restricted to participants with lower levels of psychosis. LCs were only found at level 5. These LCs were robust and largely corresponded to diagnostic categories in pre-existing glossaries, including prototypical features of schizophrenia (Class 3) and schizoaffective disorder-depressed type, or schizophrenia with a co-morbid depressive episode (Class 4). For Classes 3 and 4, the high prevalence of nuclear symptoms in each was the strongest feature supporting this classification in the absence of clinical interviews. Class 2 corresponded to a diagnosis of depressive disorder. The moderate level of associated PEs in this class would correspond to previous observations of close association of depression with PEs which increases with increasing depression severity [6–13]. Class 1 showed fewer characteristics that matched an existing diagnostic category but could be considered a sub-group with multiple PEs, possibly at risk for transition to non-affective psychosis. Alternatively, a more stable subgroup unlikely to transition and corresponding to schizotypy. This would require confirmation from longitudinal study.

### Etiological factors, linearity and extra-linearity

We investigated a range of etiological risk factors and their independent associations with PEs across the continuum. Extra-linearity, with increased association with etiological factors at the severe end of the continuum, has been described as a “quasicontinuous” relationship, explained by unmeasured moderators and multifactorial etiology where multiple factors interact with each other [1, 2, 4, 5]. Although linear increase in risk is thought associated with common mental disorder and extra-linear with psychosis [2], we found both could occur with depression and psychosis in this sample. Our findings also indicated that certain risk factors had impact on severity of both PEs and depressive symptoms across the continuum. By adjusting associations with psychosis for depression, we were able to differentiate between certain risk factors whilst showing commonality of others.

Younger age and low family income both showed positive extra-linearity but were no longer present after adjustment, suggesting the impact at the most severe end of the continuum was on depression and not psychosis. In contrast, the extra-linear association between PEs and male sex remained after adjustment, corresponding to findings that non-affective psychosis is explained by underlying differences in neurodevelopmental alterations which are more common in men [32]. Adjustment revealed associations with ethnic minority status were with depression and not PEs. Environmental impact of ethnicity on expression of psychosis may be less in China than in the USA and Europe [33, 34] but increases risk of depression.

The dose response relationships between family history of psychotic and non-psychotic disorder remained following adjustment, suggesting these proxy genetic factors impacted linearly and similarly on both depression and psychosis. It is well established that psychotic and non-psychotic disorder are genetically correlated and the effect of genetic risk factors on these disorders are non-specific [35].

Adverse childhood experiences of physical and sexual abuse and neglect each showed positive extra-linear associations which remained after adjustment for depression. Meta-analysis and systematic review suggests these factors may be a common cause of both psychotic disorders [36] and depression [37].

Urban birth showed positive extra-linearity in association with PEs, with reversal of odds of association after adjustment. Extra-linearity corresponded to a Chinese general population study where urban birth was associated with high, but not lower, PE severity, particularly among those living in urban environments [38]. Taken together, these findings suggest early, sustained urban environmental exposures are specifically associated with more severe psychosis subtypes.

A key finding was the stronger association between environmental risk factors, mainly child abuse and social adversity, with the Classes experiencing higher depressive symptoms. This is also aligned with findings on the linear and extra-linear effects of the different putative etiological risk factors. Childhood trauma has been robustly associated with a number of mental disorders, including schizophrenia, depression, anxiety and bipolar disorder [39] and it has been asserted that childhood trauma does not show a stronger association with schizophrenia than depression [40]. An underlying common mechanism is thought to be increased risk of stress-related disorders due to changes in the hypothalamic-pituitary-adrenal axis (Matheson et al. 2012). Van Nierop and colleagues [39] have suggested that childhood trauma increases likelihood of a specific admixture of affective, anxiety, and psychotic symptoms which cut across traditional diagnostic boundaries. A related hypothesis is that childhood trauma initially gives rise to affective symptoms and only later to psychotic symptoms, described as an “affective pathway” [41]. Our findings partly support this but show that these associations across the spectrum with PEs were partly attenuated when we adjusted for depression. However, it was of importance that we additionally found that the associations with childhood adversity and disadvantage did not characterize either classes 1 (High risk) or 3 (Protypical Schizophrenia), which only emerged in our highest level 5 across the spectrum. The “admixture” of symptoms due to childhood trauma [39] would therefore be supported across the first four levels in our sample. But our findings differ from previous studies in showing that when four latent classes appear at level 5, there are specific associations between child abuse and adversity and classes 2 (Depression) and 4 (Schizophrenia/Depression) but not with the other two classes, suggesting these putative etiological factors are specific for clinical categories characterized substantially by depressive symptoms.

### Strengths and limitations

Our large sample with low refusal rate allowed us to test associations with risk factors that were relatively rare. However, a high-functioning sample of university students meant we excluded important risk factors associated with poor premorbid adjustment, more likely to result in negative and disorganization symptoms and expression of non-affective psychosis [7]. Nevertheless, we still found categorical, phenotypical expression of psychosis in our sample.

Other limitations include use of self-report instruments. We did not interview participants to confirm whether those with categorical representations of psychosis actually presented with clinical psychosis. Further to this limitation, our study did not have the advantage of the survey conducted in Izmir, Turkey by Binbay and colleagues which used a clinical operationalization of the psychosis continuum [2]. Furthermore, it is probable that our most severe level 5 largely overlapped with level 4 (high impact psychotic symptoms) in the Izmir study, with few participants receiving a clinical diagnosis of psychosis.

Sample size is quite different across the 5-level PEs continuum, which may lower our statistical power because power is based on the smallest sample size in regression model. In Table 5, Classes 3 and 4 were fairly small despite the sample size with some cell sizes under 5 in some regression analyses.

Sample effects could explain lack of association between PEs and family history of severe mental disorder observed in representative community samples. In addition, we did not have information on drug misuse.

An important limitation is use of SCL-90-R to measure psychosis. Most current research defines PEs as ‘positive’ symptoms of hallucinations, delusions and thought disturbances, whereas PEs measured using SCL-90-R are mainly based on what might be classified as schizotypy. Furthermore, several items can be regarded as relational aspects of depression, such as poor self-confidence and somatization/neuroticism. Naming of the LCA classes is a subjective process and the identified classes correspond to the proposed clinical diagnoses needs to be confirmed by a clinical interview.

## Conclusions

Our study suggests that a threshold, or tipping point, exists along a spectrum of psychosis and that once a certain level of severity is reached, the nature of both psychotic and depressive symptoms and their associations with each other rapidly change their state. In this study, these changes began to manifest at level 5 and were not present at a lower levels on the continuum we had created using PEs. Clear boundaries began to emerge between PEs and depressive symptom clusters at this level of severity along a psychosis continuum but clustering of both PEs and depressive symptoms also occurred and diagnostic categories were then confirmed using LCA. We found four LCs: two LCs emerged where depressive symptoms were largely absent: Class 1 (High Risk) with moderately high PE prevalence, Class 3 with prototypic features of schizophrenia. Two additional LCs showed closely associated PEs and depressive symptoms: Class 2 with similarity to clinical depression, Class 4 with similarity to prototypical Schizoaffective disorder-Depressed type (or Schizophrenia with co-occurring depression). Etiological risk factors and their pattern of impact partly determined whether PEs were differentiated according to those on a continuum with depression or with non-affective psychosis. These factors impacted according to two differing patterns: firstly, linear impact, either across the entire continuum or until a certain level of symptom severity, representing a dimensional effect; secondly, those showing dramatic increase and disproportionate impact at the severest level. Some etiological risk factors were specific for PEs, others for depression, but most impacted on both. The pattern of results was that for PEs, proxy variables of genetic impact and loss of parent in childhood impacted mostly linearly, whereas male sex, urban birth, and child maltreatment impacted in a positive extra-linear fashion. For depression, low family income, and factors common to PEs (family history, loss of parent) impacted linearly, whereas younger age and child maltreatment (common to PEs) impacted in a positive extra-linear fashion. The extra-linear impacts of male sex and urban birth were specific to PEs and the dose-response pattern of their associations across the continuum had been obscured until adjustment for depression. Further longitudinal investigation should incorporate neuropathological measures and genetic markers together with factors we measured to further differentiate between PEs associated with depression and affective psychosis and those along a continuum with non-affective psychosis [38].

## Supplementary Information

**Additional file 1.**

## Data Availability

The datasets used and/or analysed during the current study are available from the corresponding author on reasonable request.

## Abbreviations

PEs: Psychotic experiences
PHQ-9: Patient health questionnaire-9
LCA: Latent class analysis
LMR: Lo-Mendell-Rubin
SCL-90-R: The symptom checklist-90-revised
SDs: Standard deviations
