# Supplementary material for: Testing extra-linearity across a psychosis continuum
Source: BMC Psychiatry. 2021 Nov 16;21:574. doi: 10.1186/s12888-021-03498-3 (PMC8594101; doi:10.1186/s12888-021-03498-3)
Supplement: Supplementary file 1 — Additional file 1. [file 12888_2021_3498_MOESM1_ESM.docx]

**Supplementary materials**

# Categorical psychosis measures

The following items form Symptom Checklist-90-Revised (SCL-90-R) were used in this study. **Schizophrenia Nuclear Symptoms** were defined based on item 7, 16, 35 and 62; **Hallucinations and Delusions** were defined based on item 8, 18, 43, and 83.

**Psychoticism**

7. The idea that someone else can control your thoughts

16. Hearing voices that other people do not hear

35. Other people aware of your private thoughts

62. Having thoughts that are not your own

77. Feeling lonely even when you are with people

84. Having thoughts about sex that bother you a lot

85. The idea that you should be punished for your sins

87. The idea that something serious is wrong with your body

88. Never feeling close to another person

90. The idea that something is wrong with your mind

**Paranoid ideation**

8. Feeling others are to blame for your troubles

18. Feeling that most people cannot be trusted

43. Feeling that you are watched or talked about by others

68. Having ideas or beliefs that others do not share

76. Others not giving you proper credit for achievements

83. Feel that people will take advantage of you if you let them

# Latent class analyses (LCA)

To avoid local maxima, all LCA models were estimated with different random starting values. We started with 50 random starts at the initial stage and 5 optimisations at the final stage to save computation time; if the log-likelihood could not be replicated (an indication of local maxima), we increased the parameters to 2000 and 200 respectively. Log-likelihood is the logarithm of the likelihood ratio, a test that compares the fit of two models by examining how much more likely the data are predicted by one model compared to the other. The best solution is the solution with the largest log-likelihood.

Selection of optimal number of latent classes was based on information criteria, Lo-Mendell-Rubin (LMR) test, and entropy. Information Criteria are statistics created to aid model selection by penalizing the number of factors in a model, including Akaike’s, Information Criterion (AIC), Bayesian Information Criterion (BIC) and the sample-size-adjusted BIC; lower observed values indicate better fit. Previous simulation has indicated that BIC is better than other statistics when choosing number of classes. We thus gave priority to BIC in this study. The LMR test was also used to compare models with differing numbers of latent classes (k vs. k-1), where a non-significant value (P < 0.05 rejects the k-1 model in favour of the k class model. LMR test is better than the traditional likelihood ratio chi-square test because 2 times the loglikelihood difference is not chi-square distributed. Entropy is the degree of classification accuracy of placing participants into classes based on their model-based posterior probabilities. Higher entropy values reflect better classification of individuals. We finally drew a plot of model probabilities of response for each item, where y axis is the probability that people within LC endorse a specific item (score ≥ 2).

# Continuity and discontinuity

We have customized the diagram originally presented by Binbay T. et al. to explain continuity and discontinuity in our study (Fig. S1). As we understand it, extra-linearity and linearity are subtypes of dose-response relationships. A dose-response relationship is one in which increasing levels of exposure are associated with increasing/decreasing risk of the outcome. It does not define how it changes. However, for positive extra-linearity, it changes faster at the higher end. On the contrary, for negative extra-linearity, it changes slower at the higher end.

Continuity and discontinuity in the pattern of associations were tested in our study using the following model: deviation from linearity or discontinuity is defined by a significant squared term in equation (2).

$SCL90\_trans=\log\left( SCL90 score+1 \right)$…………………………………………………….(1)

$variable=SCL90\_trans+{SCL90\_trans}^{2}+age+sex$……………………………………(2)

Variables included psychopathological, demographic and etiological factors; a non-significant squared term suggested continuity (no deviation from linearity); a negative squared term suggested a qualitatively stronger association at the lower end of the depression continuum; a positive squared term suggested a qualitatively stronger association at the higher end.


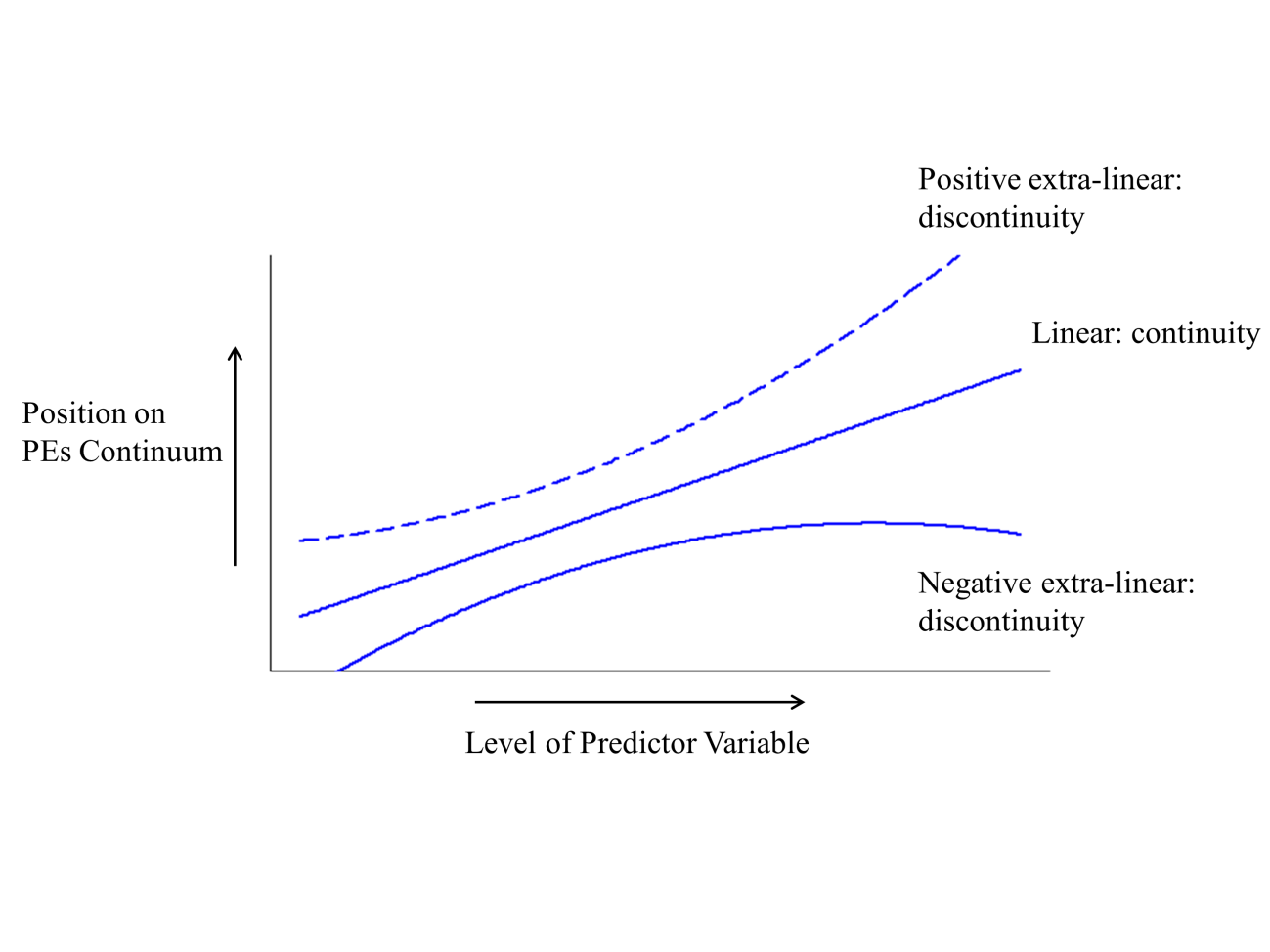


Fig. S1 Continuity and discontinuity underlying apparent continuum

A


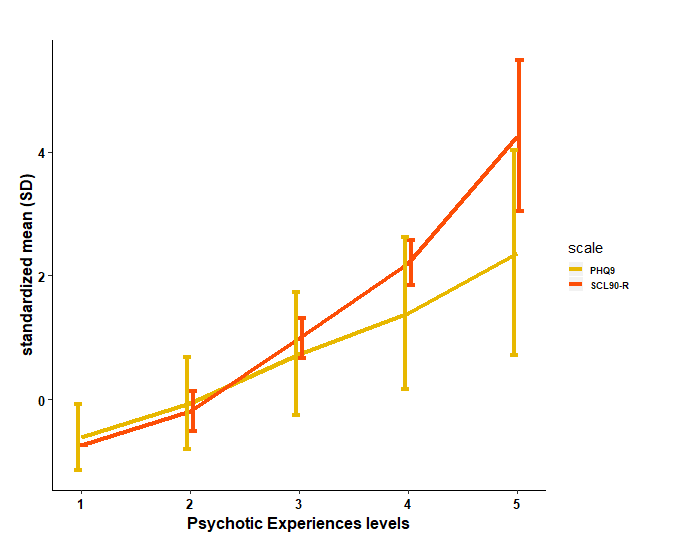


B


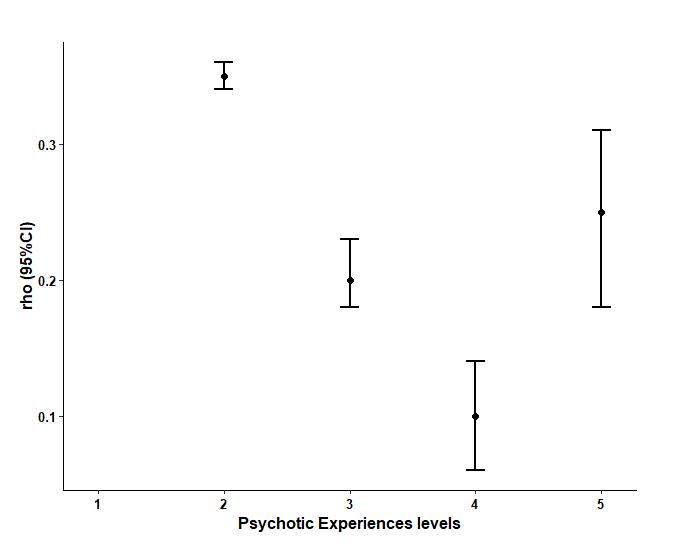


Figure S2. Descriptive representation of Psychosis and Depression measures at 5 levels with underlying item correlations (N=47,004)

A shows the mean and standard deviations for SCL-90-R and PHQ-9 at levels 1-5 across the continuum. Scores were standardized. B shows spearman’s rho correlation coefficients and 95% confidence interval between SCL-90-R and PHQ-9 at levels 2-5 across the continuum.

Table S1 Associations between Classes and SCL-90-R and PHQ9 items (n=939)

|  | Class 1  (High Risk)  N=490 (52.2%) | Class 2  (Depression)  N=337 (35.9%) | Class 3  (Prototypical Scizhophrenia)  N=51 (5.4%) | Class 4  (Schizophrenia/Depression)  N=61 (6.5%) | Class 1  **OR (95%CI)** | Class 2  **OR (95%CI)** | Class 3  **OR (95%CI)** | Class 4  **OR (95%CI)** | Class 4 vs.3  **OR (95%CI)** |
| --- | --- | --- | --- | --- | --- | --- | --- | --- | --- |
| Psychoticism |  |  |  |  |  |  |  |  |  |
| 1. Someone else controls thoughts | 209 (42.7%) | 136 (40.4%) | 44 (86.3%) | 53 (86.9%) | 1 (ref) | 0.90  (0.68-1.20) | 8.49*** (3.75-19.23) | 8.87*** (4.12-19.07) | 1.04  (0.35-3.11) |
| 16. Hearing voices others do not hear | 147  (30%) | 79 (23.4%) | 43 (84.3%) | 50  (82.0%) | 1 (ref) | 0.70*  (0.51-0.96) | 13.03*** (5.96-28.5) | 10.84*** (5.46-21.51) | 0.84  (0.31-2.31) |
| 35. Others aware of thoughts | 241 (49.2%) | 106 (31.5%) | 49 (96.1%) | 55 (90.2%) | 1 (ref) | 0.47*** (0.35-0.63) | 25.43*** (6.12-105.73) | 9.37*** (3.96-22.19) | 0.40  (0.08-2.11) |
| 62. Thoughts not your own | 306 (62.4%) | 198 (58.8%) | 51  (100%) | 59 (96.7%) | 1 (ref) | 0.86  (0.65-1.14) | n.a. | 18.53*** (4.47-76.91) | 1 (0-Inf) |
| 77. Lonely even with people | 388 (79.2%) | 320  (95.0%) | 51  (100%) | 60 (98.4%) | 1 (ref) | 4.96*** (2.90-8.46) | n.a. | 16.44** (2.25-120.36) | 0 (0-Inf) |
| 84. Bothered by thoughts about sex | 228 (46.5%) | 138 (40.9%) | 46 (90.2%) | 50  (82.0%) | 1 (ref) | 0.82  (0.62-1.09) | 11.13*** (4.31-28.76) | 5.48*** (2.75-10.91) | 0.48  (0.15-1.50) |
| 85. Should be punished for sins | 272 (55.5%) | 207 (61.4%) | 51  (100%) | 54 (88.5%) | 1 (ref) | 1.28  (0.97-1.71) | n.a. | 6.25*** (2.78-14.02) | 0 (0-Inf) |
| 87. Something serious wrong with body | 209 (42.7%) | 176 (52.2%) | 44 (86.3%) | 54 (88.5%) | 1 (ref) | 1.50**  (1.13-1.98) | 8.46*** (3.73-19.19) | 10.59*** (4.71-23.8) | 1.32  (0.42-4.13) |
| 88. Never feeling close to another person | 301 (61.4%) | 269 (79.8%) | 45 (88.2%) | 54 (88.5%) | 1 (ref) | 2.49*** (1.81-3.45) | 4.68*** (1.96-11.18) | 4.74*** (2.11-10.64) | 0.98  (0.30-3.18) |
| 90. Something wrong with mind | 229 (46.7%) | 230 (68.2%) | 47 (92.2%) | 54 (88.5%) | 1 (ref) | 2.46*** (1.84-3.30) | 13.62*** (4.83-38.4) | 9.17*** (4.08-20.63) | 0.73  (0.20-2.71) |

Table S1 (continued)

|  | Class 1  (High Risk)  N=490 (52.2%) | Class 2  (Depression)  N=337 (35.9%) | Class 3  (Prototypical Schizophrenia)  N=51 (5.4%) | Class 4  (Schizophrenia/Depression)  N=61 (6.5%) | Class 1  **OR (95%CI)** | Class 2  **OR (95%CI)** | Class 3  **OR (95%CI)** | Class 4  **OR (95%CI)** | Class 4 vs.3  **OR (95%CI)** |
| --- | --- | --- | --- | --- | --- | --- | --- | --- | --- |
| Paranoid ideation |  |  |  |  |  |  |  |  |  |
| 8. Others to blame for troubles | 280 (57.1%) | 192 (57.0%) | 51 (100%) | 52 (85.2%) | 1 (ref) | 0.98  (0.74-1.30) | n.a. | 4.34*** (2.09-9.01) | 0 (0-Inf) |
| 18. Most people cannot be trusted | 365 (74.5%) | 257 (76.3%) | 51 (100%) | 60 (98.4%) | 1 (ref) | 1.07  (0.78-1.49) | n.a. | 20.92** (2.86-152.82) | 0 (0-Inf) |
| 1. Watched or talked about by others | 317 (64.7%) | 169 (50.1%) | 50 (98%) | 59 (96.7%) | 1 (ref) | 0.54*** (0.41-0.72) | 27.35**  (3.75-199.74) | 15.96*** (3.85-66.16) | 0.58  (0.05-6.80) |
| 68. Ideas or beliefs others do not share | 333 (68.0%) | 269 (79.8%) | 48 (94.1%) | 61 (100%) | 1 (ref) | 1.88*** (1.35-2.62) | 7.91***  (2.42-25.90) | n.a. | 0 (0-Inf) |
| 1. Others do not give credit for achievements | 323 (65.9%) | 203 (60.2%) | 51 (100%) | 58 (95.1%) | 1 (ref) | 0.78  (0.59-1.04) | n.a. | 10.3*** (3.17-33.42) | n.a. |
| 1. People take advantage if you let them | 187 (38.2%) | 98 (29.1%) | 45 (88.2%) | 47 (77.0%) | 1 (ref) | 0.67**  (0.50-0.90) | 12.11*** (5.06-28.97) | 5.36*** (2.87-10.03) | 0.43  (0.15-1.24) |

Table S1 (continued)

|  | Class 1  (High Risk)  N=490 (52.2%) | Class 2  (Depression)  N=337 (35.9%) | Class 3  (Prototypical Schizophrenia)  N=51 (5.4%) | Class 4  (Schizophrenia/Depression)  N=61 (6.5%) | Class 1  **OR (95%CI)** | Class 2  **OR (95%CI)** | Class 3  **OR (95%CI)** | Class 4  **OR (95%CI)** | Class 4 vs.3  **OR (95%CI)** |
| --- | --- | --- | --- | --- | --- | --- | --- | --- | --- |
| Depression |  |  |  |  |  |  |  |  |  |
| 1. little interest of pleasure in doing things | 27 (5.5%) | 202 (59.9%) | 4  (7.8%) | 49 (80.3%) | 1 (ref) | 25.55*** (16.37-39.89) | 1.47  (0.49-4.39) | 70.49*** (33.50-148.33) | 52.49*** (15.07-182.75) |
| 2. feeling down depressed or hopeless | 39  (8.0%) | 252 (74.8%) | 6 (11.8%) | 53 (86.9%) | 1 (ref) | 35.59*** (23.51-53.87) | 1.56  (0.63-3.9) | 83.76*** (36.70-191.16) | 74.67*** (19.13-291.46) |
| 3. trouble falling or staying asleep or sleeping too much | 101 (20.6%) | 193 (57.3%) | 4  (7.8%) | 46 (75.4%) | 1 (ref) | 5.19*** (3.82-7.07) | 0.33*  (0.12-0.94) | 12.13*** (6.49-22.69) | 35.94*** (11.03-117.06) |
| 4. feeling tired or having little energy | 136 (27.8%) | 270 (80.1%) | 7 (13.7%) | 58 (95.1%) | 1 (ref) | 10.90*** (7.78-15.26) | 0.41*  (0.18-0.94) | 54.16*** (16.59-176.85) | 120.11*** (29.34-491.74) |
| 5. poor appetite or overeating | 69 (14.1%) | 197 (58.5%) | 3  (5.9%) | 45  (73.8%) | 1 (ref) | 8.60*** (6.15-12.02) | 0.38  (0.12-1.27) | 17.68***  (9.43-33.15) | 47.6***  (12.74-177.85) |
| 6. feeling bad about yourself-or that you are failure or have let yourself or your family down | 81 (16.5%) | 262 (77.7%) | 2  (3.9%) | 57  (93.4%) | 1 (ref) | 17.95*** (12.61-25.55) | 0.21*  (0.05-0.87) | 75.39*** (26.47-214.72) | 383.81*** (61.91-2379.23) |
| 7. trouble concentrating on things, such as reading the newspaper or watching television | 111 (22.7%) | 228 (67.7%) | 4  (7.8%) | 55  (90.2%) | 1 (ref) | 7.13*** (5.22-9.74) | 0.29*  (0.10-0.82) | 31.26*** (13.10-74.61) | 114.75*** (29.35-448.61) |
| 8. moving around a lot more than usual- | 56 (11.4%) | 164 (48.7%) | 2  (3.9%) | 55  (90.2%) | 1 (ref) | 7.43*** (5.22-10.56) | 0.32  (0.07-1.34) | 72.57*** (29.78-176.84) | 252.95*** (44.83-1427.24) |
| 9. Thoughts that you would be better off dead or of hurting yourself | 12  (2.4%) | 91  (27.0%) | 2  (3.9%) | 34  (55.7%) | 1 (ref) | 15.26*** (8.18-28.48) | 1.59  (0.35-7.34) | 51.00*** (23.61-110.15) | 32.20***  (7.10-146.06) |

Adjusted for age and sex
